# Mortality in a Case of Crystal Gel Ball Ingestion: An Alert for Parents

**Published:** 2012-03-01

**Authors:** Bilal Mirza, Afzal Sheikh

**Affiliations:** Department of Pediatric Surgery, The Children's Hospital and the Institute of Child Health Lahore, Pakistan

**Keywords:** Crystal balls, Jelly balls, Mortality

## Abstract

Decorative crystal gel balls are used for decoration purpose. Due to their attractive appearance they may be ingested by children. This may result in grave complications. A case of decorative crystal ball ingestion is being reported in a 6 months old infant who presented with sub acute intestinal obstruction and was operated. Crystal gel balls were causing obstruction of jejunum. Enterotomy and removal of the mass of jelly balls was done with primary closure. The patient was re-operated for anastomotic disruption on 6th postoperative day. Baby developed septicemia, and succumbed after 2 days of second operation.

## INTRODUCTION

Decorative crystal jelly balls because of their attractive features are used to decorate houses and offices. They are very small colored pellets made of superabsorbent polymer (SAP) which swell when they come in contact with water. On ingestion they can produce complications of serious nature [1,2]. Herein, we report a case of crystal ball ingestion where post operative outcome was dreadful. 

## CASE REPORT

A 6-month-old male infant presented in surgical emergency with history of bilious vomiting for 25 days and non passage of stool for a week. There was no history of abdominal distension. The patient on presentation could not tolerate feeds rather he was reluctant to take the feeds. The baby was investigated in another hospital where upper gastrointestinal contrast study showed obstruction in proximal jejunum (Fig. 1). CT scan of abdomen revealed a rounded intra-luminal mass obstructing the bowel lumen (Fig. 2). Patient had also developed jaundice Liver function tests showed, total bilirubin 3.2mg/dl, direct 1.2mg/dl, alkaline phosphatase 220IU/lit with SGPT and SGOT were within normal limits. Abdominal radiograph showed signs of sub acute intestinal obstruction.

**Figure:**
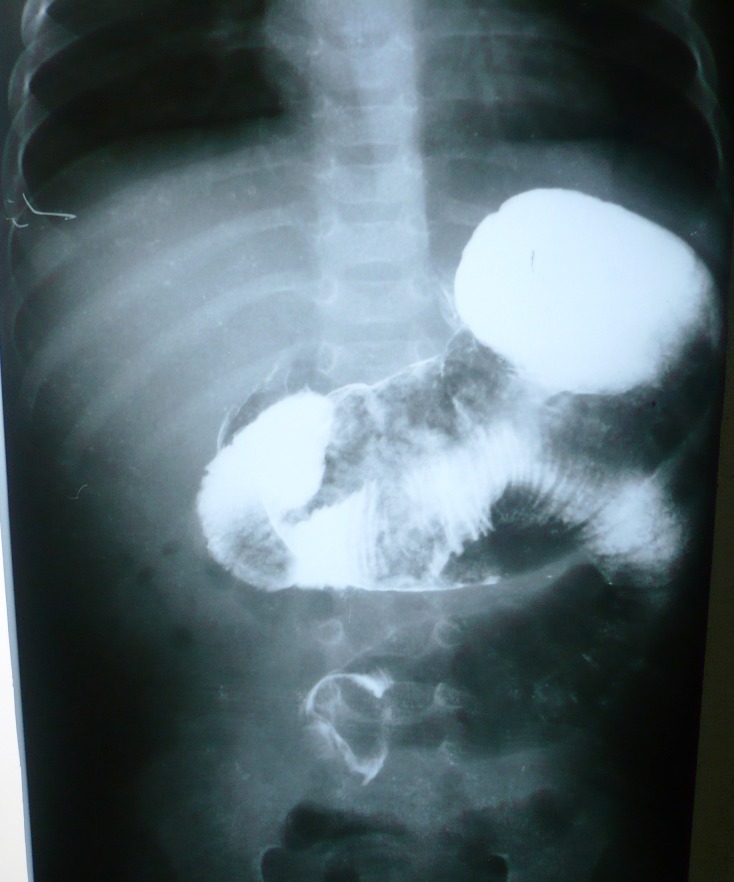
Figure 1: GIT contrast study showing obstruction and filling defect in the proximal jejunum.

**Figure:**
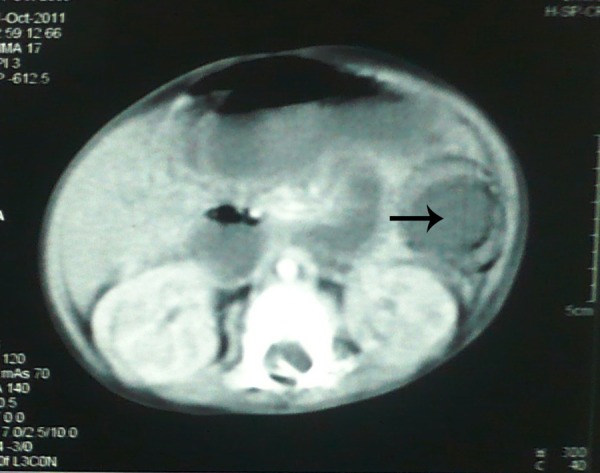
Figure 2: CT scan showing a hypo-dense intraluminal bowel mass.

Surgery was planned for sub acute obstruction intestinal obstruction. On exploration, the proximal jejunum was distended, edematous and thickened, having putty like material on palpation, being stuck and could not be moved. Enterotomy was performed which showed edematous and swollen bowel mucosa. Jelly balls formed thick sludge and caused obstruction. The jelly like material was removed from the jejunum (Fig. 3). Enterotomy was closed transversely as after retrieval of jelly material the bowel was looking healthy and not compromised. Post operatively the parents were enquired about crystal jelly balls and mother admitted that the boy from neighbors gave the baby two jelly balls and he swallowed one of them.

The patient developed burst abdomen on 6th postoperative day and was re-operated. At exploration anastomotic leak was found. The anastomosis was revised and abdomen closed by retention sutures. The patient developed septicemia, and succumbed after 2 days of second operation.

**Figure:**
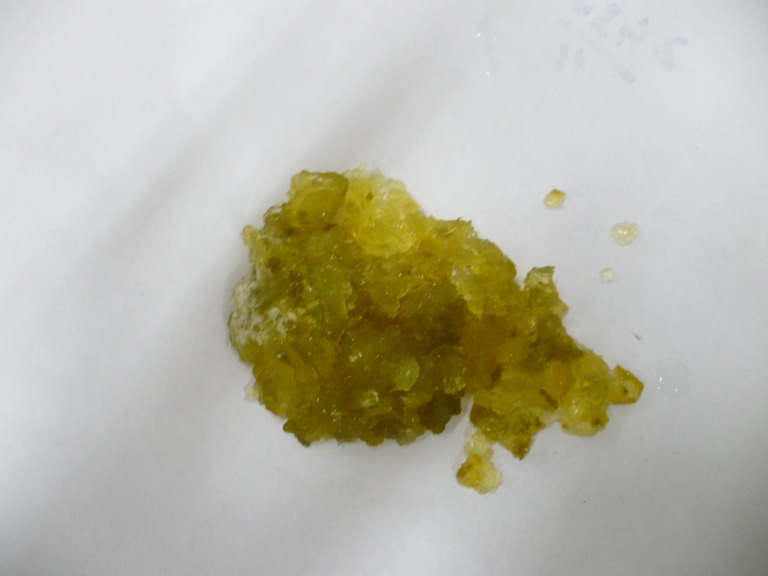
Figure 3: The retrieved crystal jelly material from small bowel.

## DISCUSSION

Decorative crystal balls, also called jelly balls or magic balls, swell when they come in contact with water or water containing solutions. Their size range between 1 and 4 mm and can absorb water 500 times of their weight and swell up to 30-60 times of their original volume. Their ingestion can lead to dreadful complications owing to their property of swelling in liquids. The complications following ingestion can range from partial or complete intestinal obstruction as in the index case to more severe form i.e. perforation peritonitis [1,2].

In the index case the patient was diagnosed about a month of ingestion of crystal ball. The ball initially caused partial intestinal obstruction followed by complete intestinal obstruction upon presentation to us. As the jelly like material was retrieved off a small enterotomy in the jejunum therefore considering it safe the enterotomy was closed transversely that then led to disruption of the repair; nevertheless in presence of jaundice and critical condition of the patient a resection and anastomosis was not opted, feared of anastomotic disruption.

Based on our experience of dealing these cases we recommend immediate endoscopic retrieval if the patient presents immediately after ingestion. Community awareness through print and electronic media is required to ban such material in the community where children can ingest this stuff.

## Footnotes

**Source of Support:** Nil

**Conflict of Interest:** None declared
